# Not all plaques are created equal: Uncovering a unique molecular signature in Alzheimer’s disease

**DOI:** 10.1177/23982128241280001

**Published:** 2024-09-20

**Authors:** Kristjan Holt, Emily Payne, Tara L. Spires-Jones

**Affiliations:** 1 Translational Neuroscience PhD Programme, The University of Edinburgh, Edinburgh, UK; 2 Centre for Discovery Brain Sciences and UK Dementia Research Institute, The University of Edinburgh, Edinburgh, UK

**Keywords:** Alzheimer’s disease, amyloid β, neuritic plaque, mass spectrometry imaging

## Abstract

Although neuritic plaques – comprised of aggregated fibrils of the misfolded protein, amyloid β (Aβ) – have formed a central focus of Alzheimer’s disease (AD) research for decades, it is now well understood that plaque burden alone is a poor correlate of cognitive decline. This is highlighted especially when compared against other neuropathological hallmarks, such as synapse loss (the strongest correlate) and hyperphosphorylated protein tau. However, it is known that Familial AD arises due to autosomal dominant mutations directly influencing the generation of Aβ, suggesting that Aβ pathology may play a key upstream role in the disease. Such contrasting lines of evidence have thus raised questions as to why some aged individuals with high plaque burden develop AD while others remain cognitively healthy. In their recent study, published in Analytical Chemistry (June 2024), Enzlein and colleagues aimed to investigate whether differences in the molecular composition of plaques between individuals with sporadic Alzheimer’s disease (N = 9) versus age-matched amyloid positive but cognitively unaffected controls (N = 8) could go towards explaining this outstanding question in the field. Using novel methods integrating mass spectrometry imaging with machine learning feature extraction, the authors compared peptide and lipid profiles to a resolving limit of 400 μm2 for >5000 individual plaques. In doing so, a distinct peptide signature was identified in sporadic Alzheimer’s disease plaques that was characterised by strongly increased aggregation of the short amyloid β isoform, Aβ1-38 coupled with a lesser co-aggregation of pyroglutamate-modified Aβ3-42pE. Sporadic Alzheimer’s disease plaques also demonstrated a robust lipid signature denoted by an increased presence of cell membrane components, GM1 and GM2 gangliosides. Here, we review this work; aiming to place these findings within the context of existing literature and with a view to discussing their importance in developing our current knowledge of Alzheimer’s disease.

***Reviewing*:** Enzlein, T., Lashley, T., Sammour, D.A. *et al.* (2024) Integrative Single-Plaque Analysis Reveals Signature Aβ and Lipid Profiles in the Alzheimer’s Brain. *Anal. Chem*, **96**(24): 9799-9807.

## Introduction

**Figure 1.**
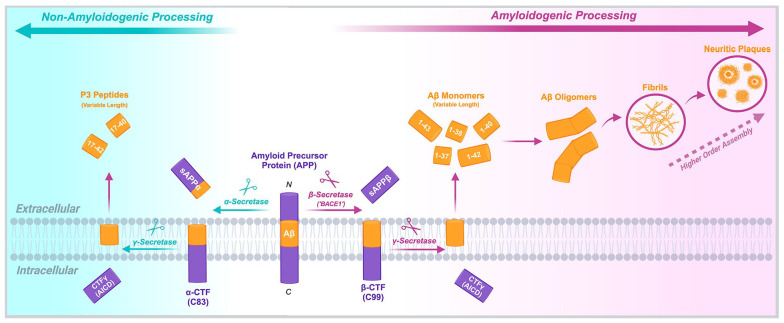
Amyloid Precursor Protein (APP) exists as a 695–770 amino acid transmembrane protein, the endogenous function of which remains poorly understood. APP is processed through sequential cleavage by secretase enzymes as part of two mutually exclusive pathways. The ‘non-amyloidogenic’ pathway (left) occurs more frequently under homeostatic conditions and involves initial cleavage by α-secretase to produce the N-terminus sAPPα and the intermediate C83 fragment. C83 is then further cleaved by γ-secretase, generating the final products of AICD and ‘P3’ peptides. Conversely, the ‘amyloidogenic’ pathway (right) involves initial cleavage by β-secretase, producing sAPPβ and C99, followed by subsequent cleavage by γ-secretase; again, generating AICD, but in this case, liberating the full-length Aβ peptide sequence as a monomer. Aβ monomers can differ in length based on their C-terminus, ranging from ‘short’ isoforms, such as Aβ_1-38_ and Aβ_1-40_ to ‘long’ isoforms, such as Aβ_1-42_ and Aβ_1-43_. These C-terminus variants are produced due to differential sites of γ-secretase cleavage. The hydrophobicity of the C-terminus – particularly in longer isoforms such as Aβ_1-42_ – increases the propensity to aggregate in the extracellular space, forming disordered cytotoxic oligomers. These then assemble into higher order structures including fibrils and β-sheeted lattices forming neuritic plaques. A shift towards amyloidogenic processing of APP and an over-production of long, hydrophobic Aβ isoforms are well-characterised drivers of plaque formation in Alzheimer’s Disease. Figure created with BioRender.com.

Neuritic plaques have remained a central focus of dementia research since their abnormal accumulation was characterised by Alois Alzheimer in his 1907 case study of ‘Auguste D’ – a 55-year-old woman who presented with symptoms of severe and progressive cognitive decline that is now known as ‘Alzheimer’s disease’ (AD). Alzheimer noted the presence of extracellular deposits he described as ‘. . . minute miliary foci which are caused by the deposition of a special substance in the cortex’. This ‘special substance’ remained elusive until technological developments in the later 20th century led to its characterisation as aberrantly processed and misfolded aggregates of the protein, ‘Amyloid-β (Aβ)’, derived from enzymatic cleavage of ‘Amyloid Precursor Protein (APP)’. In the decades since, a working model of disease emphasising APP processing remains valid to date (Figure 1).

Research has, over previous decades, often placed plaques in a central role regarding AD onset and progression. Indeed, Aβ pathology likely arises as an early event; emerging years, if not decades, prior to the onset of symptoms during the ‘clinically silent’ disease prodrome (Reiman et al., 2012). Furthermore, a clear spatial association of neuritic plaques with other pathological hallmarks such as reactive gliosis and tau-containing dystrophic neurites has begotten the logical assumption that plaques must exist as the prime upstream causative agent in AD. This thinking formed the basis for Hardy and Higgins’ ‘Amyloid Cascade Hypothesis’, which posits that plaques initiate a linear cascade resulting in the occurrence of all other AD-associated neuropathology (Hardy and Higgins, 1992). The hypothesis has, however, since become heavily disputed, due principally to frequent post-mortem observation of high plaque load in ‘cognitively resilient’ aged individuals who died without evident dementia (Kepp et al., 2023). Despite this inconsistency, the importance of plaques cannot be discarded entirely. For instance, individuals carrying autosomal dominant mutations in components of the APP processing pathway (APP, PSEN1 or PSEN2) develop a highly penetrant form of familial AD (‘FAD’), whereas elevated APP gene dosage due duplication of its locus (*21q21.3*) in Trisomy 21 results in precocious dementia-like symptoms during middle age coupled with AD neuropathological hallmarks (Lott and Head, 2019).

This has led to a major point of contention within the field: How can we reconcile what appear to be paradoxical lines of evidence for and against the role of plaques in AD? It appears evident that the Amyloid Cascade Hypothesis remains incomplete, as the mere quantity of plaques within the brain fails to explain disease onset and progression. Thus, to address this, some have turned focus instead towards investigation of differences in the *molecular composition* of plaques, themselves.

In their study, published in *Analytical Chemistry* (June 2024), Enzlein and colleagues sought to identify whether the peptide and lipid composition of plaques differed between individuals with sporadic Alzheimer’s disease (‘SAD’, N = 9) versus ‘Amyloid-positive but cognitively unaffected’ controls (‘AP-CU’; N = 8); including an additional third group of amyloid-negative individuals (‘CTRL’; N = 4). Using post-mortem tissue from the frontal cortex (Brodmann Area 9) plaques were subjected to Multimodal Mass Spectrometry Imaging (‘MSI’), capable of generating a spatial molecular map based on pixel-wise ionisation-desorption (Buchbeger et al., 2018). Combining this with machine learning (ML)-based feature extraction, the authors identified peptide and lipid features down to a resolution of 400 µm^2^.

## Key findings

By extracting ion images for Aβ_1-40_, Aβ_1-42_, and Aβ_1-38_, Enzlein *et al.* statistically identified two sub-populations of plaques stratified by their Aβ peptide composition. ‘Type 1’ plaques comprised mostly of long hydrophobic Aβ isoforms of variable N-terminus length (‘Aβ_X-42_’); were smaller in size, and more frequently appeared in AP-CU control brains. Conversely, larger ‘Type 2’ plaques were enriched for shorter C-terminus Aβ isoforms – Aβ_1-40_ and Aβ_1-38_ – and exhibited higher occurrence in SAD samples. Cross-referencing these data with colocalising features from their accompanying lipid MSI data set, the authors observed a Type 2 plaque-specific enrichment for ‘GM1’ ganglioside – a cell membrane glycosphingolipid associated with wide-ranging neuroprotective functions.

To provide unbiased support of these observations, the authors then performed unsupervised k-means clustering, which acts to sub-group (‘cluster’) unlabelled data on the basis of shared features. To achieve this, a pre-defined a range of k (the number of clusters) was tested, ranging from a minimum of ‘k = 1’ to ‘k = 10’. For each level of k, data were clustered randomly for 100 iterations, and the optimal number of clusters was determined using the Calinski-Herbasz criterion. Consequently, ‘k *=* 2’ emerged as the optimal clustering resolution, providing evidence that plaques – in the absence of any subjective experimenter influence – indeed partitioned into one of two discrete sub-groups. Multivariate statistical analysis revealed that the partitioning of plaques into these two clusters correlated strikingly with their GM content. Cluster 1 was characterised by elevated GM levels and comprised predominantly of SAD-derived plaques (1.6-fold enrichment versus AP-CU), whereas Cluster 2 demonstrated 3.3-fold enrichment in AP-CU and represented ‘low-GM’ plaques. To test the significance of these findings, the authors calculated mean Z-scores for the features of interest (total levels of Aβ isoforms and GM1-3). This in turn allowed them to then compare group means for these features between the two clusters via unpaired Student’s *t*-test. In line with the differences in fold enrichment previously identified, Aβ_1-38_ and all GM species indeed exhibited a statistically significant elevation in cluster 1 (p < 0.001).

Finally, to investigate whether Aβ & GM plaque profiles could be used to reliably distinguish SAD from AP-CU, Enzlein *et al.* incorporated an ML approach. Implementing three model architectures in parallel, ‘SHAP’ (Shap Additive exPlanations) values were calculated to identify the relative importance of the previously identified plaque features in distinguishing between SAD and AP-CU cases. An increased presence of the short isoform, *Aβ_1-38_*, was identified by all models as the single most important feature distinguishing SAD from AP-CU. Furthermore, N-terminus truncated, pyroglutamate-modified isoform *Aβ_3-42pE_* – target of recent Food and Drug Administration (FDA)-approved therapy, *Donanemab* – also appeared consistently as one of the top five most important features for defining SAD. Meanwhile, for GM species, all three models highlighted *GM1(36:1)* and *GM2(36:1)* as major differentiators that were enriched in SAD versus AP-CU. Altogether, this approach thus confirmed that the differential features highlighted from previous subjective (visual) and objective (*k*-means clustering) methods were, when taken in isolation, sufficient to reliably classify a given (‘unknown’) sample as having been derived from an SAD or AP-CU case.

## Discussion

**Figure 2.**
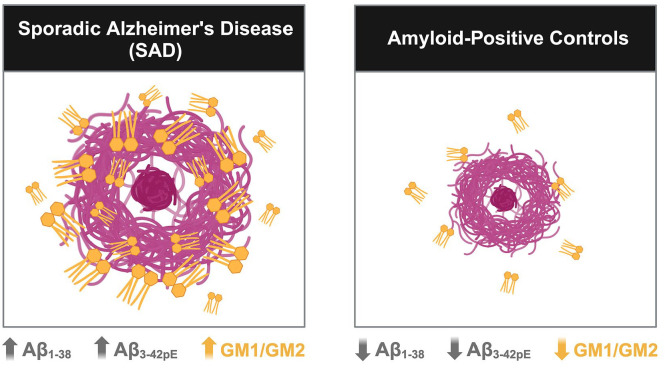
Enzlein *et al.* report that plaques derived from SAD frontal cortices (BA9) exhibit a larger size; greater accumulation of short Aβ isoforms – in particular Aβ1-38 – and selective co-aggregation of GM1 and GM2 gangliosides in the lipid fraction. Conversely, plaques from age-matched controls with no evident symptoms of dementia appear smaller and lack enrichment for short Aβ isoforms and GM gangliosides. This observation prompts further questions as to whether ‘SAD’ plaques therefore possess unique molecular properties that are distinct from ‘non-AD’ plaques, thus tentatively providing some explanation as to why some individuals appear to retain cognitive function in light of high plaque burden. Figure created with BioRender.com.

Enzlein *et al.* report differences in plaque composition between individuals with ‘SAD’ versus age-matched plaque-carrying control cases. Plaques derived from the SAD frontal cortex carry a unique peptide signature of Aβ_1-38_ and, to a lesser extent, Aβ_3-42pE_. This peptide composition appears coupled with a unique lipid profile comprised of ‘GM’ ganglioside species (Figure 2).

Although the findings of this study pose interesting avenues for further investigation, questions remain as to the biological significance of said findings when taken alone. The authors posit that the accumulation of shorter Aβ isoforms represents a later event regarding plaque formation in SAD, encircling an Aβ_1-42_ enriched core. However, this was not demonstrated directly, relying instead on citation of prior work involving a triple knock-in mouse model of aggressive amyloidosis, *APP NL-G-F*. Such models more closely recapitulate the exceptionally rarer autosomal dominant forms of FAD; themselves exhibiting an aetiology that is driven principally by APP misprocessing. This is distinct from SAD, which is hypothesised to alternatively involve impaired mechanisms of Aβ clearance (Nalivaeva and Turner, 2019). One may therefore question the extent to which observations from transgenic models can be extrapolated to the SAD population, particularly if FAD and SAD may occur as two distinct disease processes with a convergent phenotype.

Furthermore, an apparent link between Aβ_1-38_ and FAD is not new. Previous studies have demonstrated, via immunohistochemistry (IHC), a parenchymal accumulation of Aβ_1-38_ in plaques that appears unique to FAD. In SAD, most Aβ_1-38_ appears in fact to be localised vascularly as cerebral amyloid angiopathy (CAA) (Reinert et al., 2013). As the authors acknowledge the MSI limitation of 400 µm^2^ pixel area in this study, the possibility cannot be excluded that confounds may have arisen from the presence of CAA in these cases, which is more likely to be detected using such large regions of interest. Without further interrogation of tissue derived from the cases in this study – for instance, via IHC to visualise the ratio of vascular:parenchymal Aβ_1-38_ – it remains unclear as to how the findings reported here may be interpreted in the context of extant conflicting literature. In addition, the authors stop short of explaining how these findings may inform our wider understanding of AD. Indeed, it has been suggested that *increased* cerebrospinal fluid (CSF) levels of Aβ_1-38_ represent a potential biomarker to indicate a slower rate of cognitive decline (Cullen et al., 2022). As a shift from short- to long-Aβ isoform generation is a well-established process in AD, increased CSF Aβ_1-38_ can be interpreted as a proxy for ‘healthy’ γ-secretase function (Szaruga et al., 2017). Without further work to validate the authors’ findings, it is difficult to explain *why* plaques derived from SAD cases may be differentiated by their increased Aβ_1-38_ content.

A novel finding of this study was that of a plaque-associated ‘GM’ ganglioside (GM1 and GM2) signature in SAD. Interestingly, prior studies have demonstrated an apparent strong binding of oligomeric Aβ to GM1 following microinjection of the former into a healthy mouse brain (Hong et al., 2014). As GM1 exists as a cell membrane component, it is tempting to speculate that this oligomeric Aβ, which is typically present at high concentrations within the plaque halo, may interact with tau-containing dystrophic neurites that also localise to this region. The latter poses an interesting avenue for further interrogation, as tau – not Aβ – presents a stronger correlate of cognitive decline in AD (Boccalini et al., 2024). However, the mere presence of GM gangliosides in SAD plaques alone does not provide a biological explanation as to what may differentiate the SAD brain from aged controls. It could be the case that gangliosides are a mere bystander in the disease process, existing as remnants of plaque-associated neuronal, microglial, and/or astrocytic membranes in the toxic plaque microenvironment.

It is hence difficult to interpret what this study adds to our knowledge regarding AD without translation of these results into in-depth experiments. This is perhaps true of many sequencing-based studies: while their hypothesis- and data-generating potential cannot be denied, they often conclude with an optimistic refrain that they have identified novel targets for further study. While a comprehensive follow-up of their results is likely beyond the scope of this study, an acknowledgement by the authors of the work left to be done to carry their observations into useful applications would go far in appropriately contextualising their findings.
